# UHPLC-ESI-MS/MS Quantification of Relevant Substrates and Metabolites of the Kynurenine Pathway Present in Serum and Peritoneal Fluid from Gastric Cancer Patients—Method Development and Validation

**DOI:** 10.3390/ijms22136972

**Published:** 2021-06-28

**Authors:** Ilona Sadok, Katarzyna Jędruchniewicz, Karol Rawicz-Pruszyński, Magdalena Staniszewska

**Affiliations:** 1 Laboratory of Separation and Spectroscopic Method Applications, Centre for Interdisciplinary Research, Faculty of Science and Health, The John Paul II Catholic University of Lublin, Konstantynów 1J, 20-708 Lublin, Poland; ilona.sadok@kul.pl (I.S.); k.jedruchniewicz@umcs.pl (K.J.); 2 Department of Surgical Oncology, Medical University of Lublin, Radziwiłłowska 13, 20-080 Lublin, Poland; karolrawiczpruszynski@uml.edu.pl

**Keywords:** tryptophan metabolism, kynurenine pathway, gastric cancer, targeted metabolomics, LC-MS/MS, serum analysis, peritoneal fluid analysis

## Abstract

Metabolites and enzymes involved in the kynurenine pathway (KP) are highly promising targets for cancer treatment, including gastrointestinal tract diseases. Thus, accurate quantification of these compounds in body fluids becomes increasingly important. The aim of this study was the development and validation of the UHPLC-ESI-MS/MS methods for targeted quantification of biologically important KP substrates (tryptophan and nicotinamide) and metabolites(kynurenines) in samples of serum and peritoneal fluid from gastric cancer patients. The serum samples were simply pretreated with trichloroacetic acid to precipitate proteins. The peritoneal fluid was purified by solid-phase extraction before analysis. Validation was carried out for both matrices independently. Analysis of the samples from gastric cancer patients showed different accumulations of tryptophan and its metabolites in different biofluids of the same patient. The protocols will be used for the evaluation of tryptophan and kynurenines in blood and peritoneal fluid to determine correlation with the clinicopathological status of gastric cancer or the disease’s prognosis.

## 1. Introduction

Gastric cancer (GC) is the fifth most common malignancy and the third leading cause of cancer death in males and females combined worldwide (5.7% and 8.2% of the total cases for incidence and mortality, respectively, according to GLOBOCAN 2018 data). Many factors may be involved in the causation of GC, including tobacco smoking, diet and nutrition, *Helicobater pylori (H. pylori*) infection, heavy alcohol intake, and host genetic susceptibility. Avoiding consumption of red meat and preserved meat, salt, salty and smoked foods, and increasing the intake of fruits, citrus fruit, and green and yellow vegetables might significantly reduce the risk of GC [1]. GC is more likely to be diagnosed in males than females [2]. In 2018, the highest incidence rates of GC in Eastern Asia (32.1% for men and 13.2% for women), Eastern Europe (17.1% for men and 7.5% for women), South America (12.7% for men and 6.9% for women), and Western Asia (11.3% for men and 6.0% for women) were observed [3]. Although surgery is the standard treatment for GC, early detection and diagnosis significantly improves the survival rate [4].

**Figure 1:**
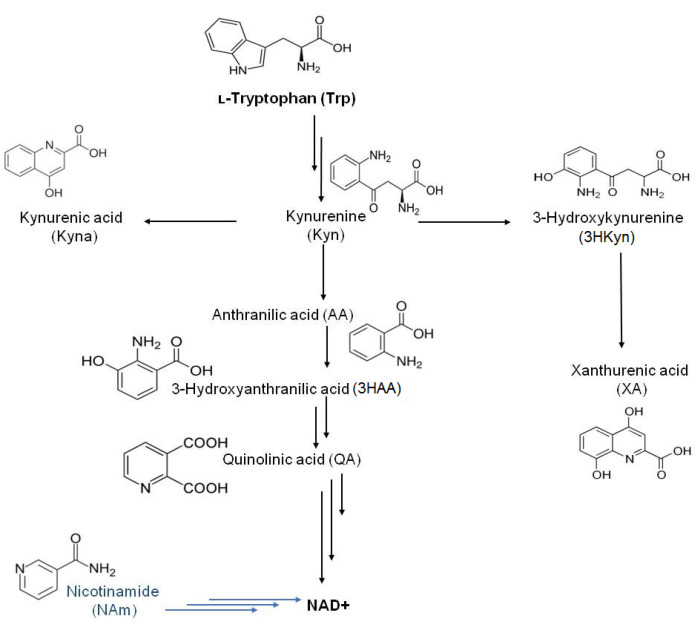
Biomolecules covered in this work.

Tryptophan (Trp) depletion via the kynurenine pathway (KP) is one important mechanism contributing to tumor immune surveillance, and it is a promising target for immunotherapy in a variety of cancers including the gastrointestinal tract [5]. This metabolic route is initiated by activation of the indoleamine 2,3-dioxygenase 1 (IDO1) enzyme, but other enzymes—indoleamine 2,3-dioxygenase 2 (IDO2), or tryptophan 2,3-dioxygenase (TDO)–also contribute to this process. IDO1 is widely expressed across different tissues and cells types (e.g., the colon, epididymis, dendritic cells, macrophages, reticular cells, and cancer cells) [6,7]. IDO2 was also found in several tissues (e.g., the brain, liver, kidney, epididymis, dendritic cells, and B cells) [6], while TDO is restricted to the liver [8]. Kynurenine pathway metabolites (Figure 1) are important factors in the regulation of the immune system and originate from Trp decomposition to N-formylkynurenine, which is rapidly converted into kynurenine (Kyn). Subsequently, several metabolites like Kyn, 3-hydroxykynurenine (3HKyn), 3-hydroxyanthranilic acid (3HAA), and quinolinic acid (QA) lead to the formation of NAD+ as the final KP product [9]. Trp metabolites exert multiple effects and interact with cellular receptors. Kyn binds to the human aryl hydrocarbon receptor (AhR) [10] and suppresses allogeneic T-cell proliferation [11]. Kyna acts as a negative allosteric modulator at the α7-nicotinic receptor and as an agonist at an orphan G-protein-coupled receptor (GPR35) and the AhR [9]. The 3HKyn metabolite induces cell death and contributes to cataract formation, but it might also work as an antioxidant, while 3HAA causes toxicity in neuronal cultures and protein damage but can also act as an inflammatory and neuroprotector molecule [12]. Xanthurenic acid (XA), which is formed from 3HKyn, leads to apoptosis of the epithelial cells in lenses and shows antioxidant and vasorelaxation properties [13,14]. QA exerts neurotoxic effects by several mechanisms [15]. Nicotinamide (NAm), which also contributes to NAD+ formation by the salvage pathway, has a similar effectiveness to vitamin B_3_ and might be used in psychiatric disorder treatment [16,17].

IDO1 expression in cancer tissue has been linked to the progression of different types of GC and can predict overall survival [18,19,20]. Larger tumor size, disease progression, and poorer prognosis are correlated with IDO1 expression in GC patients [20]. Recently, high IDO1 expression and l-Kyn presence were confirmed to promote both gastric cancer cell growth and migration ability by increasing extracellular matrix expression, especially the COL12A1 gene [21]. Other works focused on the correlation between *H. pylori* infection and the Trp and Kyn levels in the serum or plasma from GC patients [22,23]. Based on the obtained results, Trp and Kyn have been identified as potential GC biomarkers [22]. Choi et al. [24] evaluated Trp depletion in serum and gastric juice by the determination of selected metabolites from different metabolic pathways. The authors have found that Trp metabolism via the kynurenine pathway is activated in GC, and the estimation of Kyna in the serum and gastric juice might serve as diagnostic test. All of the above show KP metabolites as promising diagnostic and therapeutic targets in gastric cancer. However, the pathogenic mechanisms of IDO1 and the role of kynurenines in GC need to be further explored.

Liquid chromatography–tandem mass spectrometry (LC-MS/MS) is a well-recognized and powerful analytical technique in metabolomics. LC-MS/MS has already found broad application for the quantification of Trp-derived compounds from different metabolic routes and other bioactive compounds in samples of different origins [24,25,26,27,28,29]. The objective of this study was to develop and validate methods employing ultra-high performance liquid chromatography–electrospray ionization–tandem mass spectrometry (UHPLC-ESI-MS/MS) for Trp and KP metabolite determination in two distinct physiological matrices (serum and peritoneal fluid) collected from patients with GC. These methods employ an internal standard strategy and allow for the simultaneous quantification of Trp and six metabolites in blood serum and for Trp and eight related compounds in peritoneal fluid. The metabolites determined in this work are presented in Figure 1. The developed methodology can be used to study KP activation by analysis of serum and peritoneal fluid in relevant diseases, including GC. The protocols might also be useful for assessment of the applicability of KP metabolites as GC biomarkers in disease staging.

## 2. Results

### 2.1. Liquid Chromatography and Mass Spectrometry

**Figure 2:**
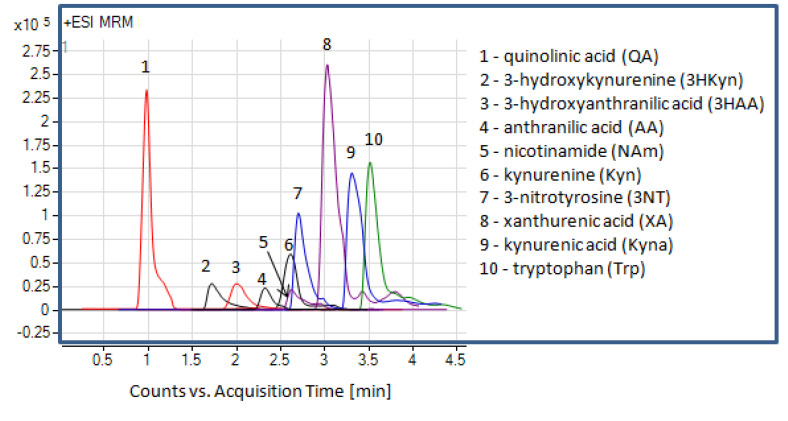
Overlaid monitored transitions selected for quantification of the target analytes (standards prepared in neat solvent).

The mass spectrometer (MS) parameters were optimized by injection (5 µL) of standards (1 g/L) prepared in neat solvents by direct application to the detector (no column). For each analyte (QA, NAm, 3HAA, AA, 3HKyn, Kyn, XA, Kyna, Trp, and 3NT), the most abundant ion on the scan ion spectra were selected as a precursor ion. The studies were performed in positive and negative ion scan modes. The scan spectra were acquired for various mobile phases to ensure high detection sensitivity for each target analyte. We tested 18 mobile phases composed of solvent A (water containing various amounts of formic acid, acetic acid, ammonium formate, or ammonium acetate) and solvent B (MeOH). Solvent A was 0–20 mmol/L ammonium formate, ammonium acetate (without or with the addition of proper acid to reach pH 4.3), or water containing formic or acetic acid (pH 4.3). The mobile phase flow rate was 0.5 mL/L (50% solvent B). The analysis of acquired data showed the abundance of the protonated ions ([M + H]^+^) to be higher than the deprotonated ions ([M − H]^−^), with 3HAA and AA being exceptions. For qualitative purposes, we decided to monitor all ions in the positive mode. To select the optimal mobile phase for simultaneous quantification of Trp and the selected metabolites, we excluded mobile phases for which at least one analyte did not generate an MS signal. The MS response of 3NT (tested as an internal standard) was also taken into consideration. Based on the obtained results, we selected a mobile phase composed of 5 mmol/L ammonium acetate (solvent A) and MeOH (solvent B). Subsequently, the optimization of the fragmentor voltage (FV) to maximize the precursor ion ([M + H]^+^) intensity was performed for each analyte in the range from 40 to 110 V, followed by optimization of the collision energy (CE) to maximize the product ion intensity (studied range: 4–30 eV). Finally, we selected two ion transitions producing the highest MS signals for the multiple reaction monitoring (MRM) method. The most intense transitions were chosen for quantification. The precursor ion and product ion (chosen for the quantification and qualification) values of the FV and CE are listed in Table 1. Then, working with the column and MRM mode, we adjusted the mobile phase gradient program to obtain satisfactory separation of the analytes within a short analysis time. The data obtained on the retention times of the analytes allowed for setting up the dynamic MRM (DMRM) method for quantitative purposes (Table 1). The overlaid MRM chromatograms for the target analytes acquired in our chromatographic system are presented in Figure 2.

### 2.2. Optimization of the Protocol for Serum Preparation

**Figure 3:**
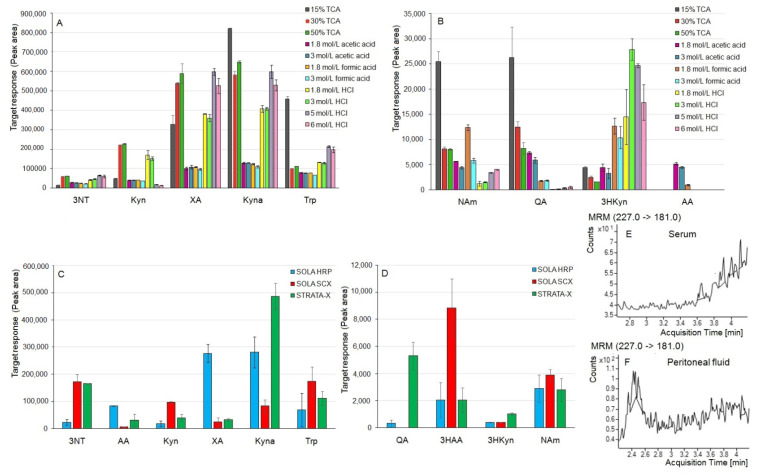
Results for optimization of the serum (**A**,**B**) and peritoneal fluid preparation (**C**,**D**) for UHPLC-ESI-MS/MS analysis. (**A**,**B**) Effect of different solvents used for protein precipitation from the serum on the MS response of the target compounds. Serum (100 µL) fortified with analytes (0.5 mg/L each) was mixed with 50 µL of the precipitating solvent, which was centrifuged (14,000× *g*, 15 min, 4 °C), and the supernatant was transferred into a chromatographic vial and analyzed by the UHPLC-ESI-MS/MS method (injection 10 µL, 3 replicates). (**C**,**D**) Comparison of signals of analytes determined in the peritoneal fluid purified on different SPE cartridges. Applied SPE conditions: (1) STRATA-X cartridge: conditioning 1 mL MeOH, equilibration 1 mL water, loading 500 µL sample, washing 1 mL 5% (*v*/*v*) MeOH in water, elution 1 mL MeOH; (2) SOLA HRP cartridge: conditioning 500 µL MeOH, equilibration 500 µL water, loading 500 µL sample, washing 500 µL 5% (*v*/*v*) MeOH in water, elution 500 µL MeOH; (3) SOLA SCX cartridge: conditioning 500 µL MeOH, equilibration 500 µL 1% (*v*/*v*) formic acid in water, loading 500 µL sample, washing 500 µL 1% (*v*/*v*) formic acid in water followed by 500 µL 1% (*v*/*v*) formic acid in MeOH, elution 500 µL 5% (*v*/*v*) ammonium hydroxide in MeOH. The eluate was gently evaporated to dryness, redissolved in 60 µL of 5 mmol/L ammonium acetate, and analyzed by UHPLC-ESI-MS/MS (injection 5 µL, 3 replicates). Before SPE, peritoneal fluid (200 µL) spiked with analytes (12.5 mg/L 3NT, 1.25 mg/L AA, 3HAA, 3HKyn, NAm, Kyn, Trp, XA, 0.5 mg/L QA, Kyna) was mixed with 800 µL of ice-cooled 2% (*v*/*v*) acetic acid in MeOH and then centrifuged (14,000× *g*, 15 min, 4 °C). For data interpretation, values obtained for the control (peritoneal fluid without spike with targets) were subtracted from those for the fortified samples. The figure presents the mean values from two independent experiments. (**E**,**F**) LC–MS/MS chromatograms for MRM transition (227 > 181), selected for 3NT in the blank serum (**E**) and peritoneal fluid (**F**) from gastric cancer patients.

The serum samples were prepared by simple protein precipitation before chromatographic analysis. To select the best conditions for simultaneous Trp, kynurenines, and NAm determination, we tested different solvents for protein removal. First, we investigated the effect of common organic solvents (MeOH, ACN, and acetone) with or without 0.1% formic acid (*v*/*v*) on the MS response of the target analytes (QA, NAm, 3HAA, AA, 3HKyn, Kyn, XA, Kyna, Trp, and 3NT), maintaining a 1:1 (*v*/*v*) serum-to-solvent ratio. Our previous observations on the post-culture media from human cancer cells [30] indicated that using an acidified organic solvent for protein precipitation might improve the recovery of kynurenines, determined in biological samples. In this work, serum (fortified with known amounts of analyte standards) was mixed with an ice-cooled precipitating solvent, centrifuged, evaporated to dryness, and reconstituted in 5 mmol/L ammonium acetate. Although analytes were detectable in the serum after protein precipitation, all examined crashing solvents resulted in a small amount of precipitate. Since ACN was found to be the most efficient precipitating agent, we increased the ratio of serum to ACN up to 2:1 (*v*/*v*). However, no improvement in protein precipitate formation was observed. To avoid analytical column clogging and LC-MS/MS system contamination, we decided to test stronger precipitating agents. The results for ACN containing from 0% to 50% (*v*/*v*) of 50% (*w*/*v*) TCA used as the precipitating agent (serum-to-solvent ratio of 2:1, *v*/*v*) were compared. The addition of TCA into ACN resulted in significant loss of 3HAA (about 90–95% signal decrease compared with pure ACN) and AA (signal disappeared for ACN containing 25% (*v*/*v*) of 50% (*w*/*v*) TCA). On the other hand, the presence of TCA improved the MS response of QA, Trp, Kyn, Kyna, and XA; however, the efficacy of protein removal was still insufficient. Finally, we studied the efficacy of protein precipitation by aqueous solutions of common acids (15–50% (*w*/*v*) TCA, 1.8–6 mol/L HCl, 1.8–3 mol/L formic acid, and 1.8–3 mol/L acetic acid) at different concentrations on the MS signals of the target analytes (Figure 3A,B). Aqueous 1.8–3 mol/L acetic acid or formic acid yielded the lowest amount of precipitate, followed by HCl, whereas TCA was found to be the most effective precipitating agent. The best 3HKyn recoveries were noted after protein removal by HCl-based mixtures (the best results being for 3 mol/L HCl), probably due to the good dissolution of this analyte in this type of solvent. AA was only detected in the acetic acid solutions and 1.8 mol/L formic acid. The 3HAA signal disappeared after protein removal by all tested solvents. Considering both the amounts of precipitate and the recoveries of the target analytes, we selected a 15% (*w*/*v*) aqueous solution of TCA for protein removal from the serum. Furthermore, AA and 3HAA were excluded from the list of analytes that were simultaneously determined in the serum and were not subjected to the method validation procedure. Moreover, 3NT was not found in the blank serum sample (Figure 3E), proving the applicability of the chosen internal standard.

### 2.3. Development of the Protocol for Peritoneal Fluid Preparation

At first, we tested the utility of the simple removal of proteins and cellular material (e.g., from blood contamination) by precipitation with organic solvents (MeOH and ACN) with or without the addition of acetic acid or formic acid (0–2%, *v*/*v*) before UHPLC-ESI-MS/MS analysis. After precipitation, the samples (peritoneal fluid spiked with standard analytes (200 µL) mixed with the organic solvent (800 µL)) were centrifuged, and the supernatants were transferred into glass vials, evaporated to dryness, reconstituted in 5 mmol/L ammonium acetate (100 µL), and analyzed by UHPLC-ESI-MS/MS. Each experiment was repeated twice, and a mean value was used for results comparison. The analytes selected for method development (QA, NAm, 3HAA, AA, 3HKyn, Kyn, XA, Kyna, Trp, and 3NT) showed differences in extraction yields depending on the solvent type. The 3HKyn was found to be the most problematic due to a significant loss during the sample preparation step, regardless of the applied experimental conditions. The solvents acidified with acetic acid provided better or similar results compared with the solvents containing formic acid. Furthermore, no significant differences in recoveries were noted between the MeOH-based and ACN-based solvents. MeOH containing 2% (*v*/*v*) acetic acid seemed to provide the most optimal conditions for the simultaneous determination of several selected analytes and was further evaluated. Unfortunately, the analytical column pressure was systematically increasing after sample injections, and sample nebulization caused strong contamination with a white coating of external parts of the MS ion source, suggesting an insufficient pretreatment of the peritoneal fluid. To overcome this problem, we decided to implement SPE for the sample pretreatment.

Different cartridges were tested for SPE: Strata-X (containing a surface modified styrene divinylbenzene polymer as a sorbent, suitable for the extraction and purification of both polar metabolites and less polar parent compounds), SOLA HRP (reversed phase), and SOLA SCX (mixed-mode strong cation exchanger). During the preliminary studies, the peritoneal fluids spiked or not spiked (control) with standard analytes were diluted five times with 2% (*v*/*v*) acetic acid in MeOH and centrifuged, and the supernatants were subjected to SPE, adopting conditions recommended by the vendor (see description of Figure 3). Importantly, the control (peritoneal fluid without added standards) did not contain 3NT (selected as the internal standard; Figure 3F). The best LC-MS/MS signals were obtained after sample purification on SOLA HRP for AA and XA, on SOLA SCX for 3HAA, Kyn, and Trp, and on STRATA-X for QA, 3HKyn, and Kyna (Figure 3C,D). For further optimization steps, we selected the STRATA-X cartridges, considering the promising results for QA and 3HKyn (which showed low ionization efficiency) and acceptable data for other targets.

The SPE purification step was further optimized using a representative sample of peritoneal fluid (obtained by pooling material from five different patients) fortified or not fortified (control) with the known amounts of the targets. We investigated the impact of the solvent used for sample dilution before SPE on the MS signals of the targets. Peritoneal fluid (400 µL) was mixed with 500 µL of 2% (*v*/*v*) acetic acid in MeOH or water containing from 0% to 5% (*v*/*v*) acetic acid. Water-based solvents resulted in higher recoveries of all analytes compared with acidified MeOH. The presence of acetic acid in water improved the recoveries of 3HAA, XA, Kyn, Kyna, Trp, and 3NT, but in a different manner. Water acidification had no impact on the 3HKyn recoveries. Only in the cases of QA, AA, and NAm was a decrease of recoveries noted after acetic acid addition. Based on the obtained results, the peritoneal fluid was mixed with 1% (*v*/*v*) acetic acid in water before loading on a STRATA-X cartridge. Next, the washing step was optimized. After sample loading, the SPE cartridges were washed using water containing 0%, 2%, or 5% (*v*/*v*) MeOH. Increasing the MeOH concentration in the washing solvent increased the analyte loss, and thus washing with water was chosen. Finally, we tested different solvents for sample elution (MeOH, MeOH:ACN (1:1, *v*/*v*), MeOH:ACN:HCl (1 mol/L) (40:40:20, *v*/*v*/*v*), MeOH:ACN:HCl (3 mol/L) (40:40:20, *v*/*v*/*v*), MeOH:ACN:HCl (6 mol/L) (40:40:20, *v*/*v*/*v*), MeOH:ACN:acetic acid (2 mol/L) (40:40:20, *v*/*v*/*v*),andMeOH:ACN:acetic acid (3 mol/L) (40:40:20, *v*/*v*/*v*)). The best recoveries for 3HAA and AA were obtained using a solvent containing 1 mol/L HCl, while for 3HKyn and XA, it was a solvent containing 6 mol/L HCl, and for 3NT, QA, NAm, Kyn, and Kyna, it was solvent containing 3 mol/L acetic acid. Meanwhile, for Trp, it was a solvent containing 2 mol/L acetic acid. It should be noted that all the tested solvents composed of HCl caused serious loss of NAm during sample elution. Finally, elution with MeOH:ACN:acetic acid (3 mol/L) (40:40:20, *v*/*v*/*v*) was selected as the most optimal approach.

### 2.4. Method Validation

The developed methods for serum and peritoneal fluid were validated according to the general requirements specified in the FDA guideline [31].

The linearity range for each selected analyte was established using the calibration curves prepared with the serum and peritoneal fluid. Analyte-free matrices were obtained by pretreatment with activated charcoal. Each calibration plot contained at least six points. All working calibration curves had the acceptable coefficient of determination (R^2^ > 0.990) within the tested ranges for both matrices studied (Table 2). The linear ranges covered concentrations in the range from a low amount of nmol/L to tens of µmol/L for all analytes tested in both matrices. The LOQs were in the order of a low amount of nmol/L for the studied metabolites. Detailed data are collected in Table 2.

The interday and intraday accuracy were excellent for all target compounds (not exceeding ±20% at LOQ and ±15% at other concentration levels) in both the serum and peritoneal fluid. Additionally, all data obtained for the interday and intraday precision met the established criteria (CV ±20% at LOQ and ±15% at other concentration levels). The precision and accuracy results of the established methods for the serum and peritoneal fluid are included in Table 3.

The analytical recoveries were from 81.44% to 103.62% for the serum. The recoveries in the peritoneal fluid were typically >82%, with 3HKyn (49.39–57.89%) being the exception (Table 4).

Coeluting the compounds from the serum and peritoneal fluid matrices differently influenced ionization of the target analytes. These effects were expressed as a matrix effect. In the serum, ion suppression was observed in the cases of Trp, XA (for different studied concentrations), and 3HKyn (at the LOQ concentration level). The highest ion enhancement was noted for NAm (about 154.05% for the highest concentration studied). For other analytes, no serious matrix effects were observed in the serum. The highest ion suppression in the peritoneal fluid was noted for QA, 3HKyn, AA, and 3HAA (73.90–89.90%) for the different concentrations studied. This matrix was found to slightly enhance Kyn (121.38–123.75%) and NAm (107.11–128.01%) ionization, whereas the matrix effects for the other analytes were negligible. No serious matrix effect for 3NT was observed for both the studied matrices. The detailed results are presented in Table 4.

Some analytes were found to be unstable and undergo significant degradation in the presence of the sample matrix components. In the serum, 3HKyn showed poor stability over 24 h at room temperature (degradation up to 53.74–76.28% when stored in an autosampler) and after freezing and thawing (about 30% loss). In the case of Trp, no more than 20% degradation was observed when stored for 24 h in an autosampler and after freezing and thawing. The remaining analytes were considered stable in the presence of the serum matrix under the tested conditions. In the case of the peritoneal fluid matrix, up to 20% degradation of 3HKyn when stored in an autosampler for 24 h was noted. Significant loss in 3HKyn was observed after sample freezing and thawing (about 30–35%, depending on the evaluated concentration level). For XA, up to 20% degradation could be observed after 24 h of storage in an autosampler or sample freezing and thawing. Storage in an autosampler of the peritoneal fluid sample promoted 3HAA and 3NT degradation (up to 34% and 20%, respectively). The other analytes were found to be stable in the peritoneal fluid under the studied conditions (Table 4).

### 2.5. Application to Clinical Samples

**Figure 4:**
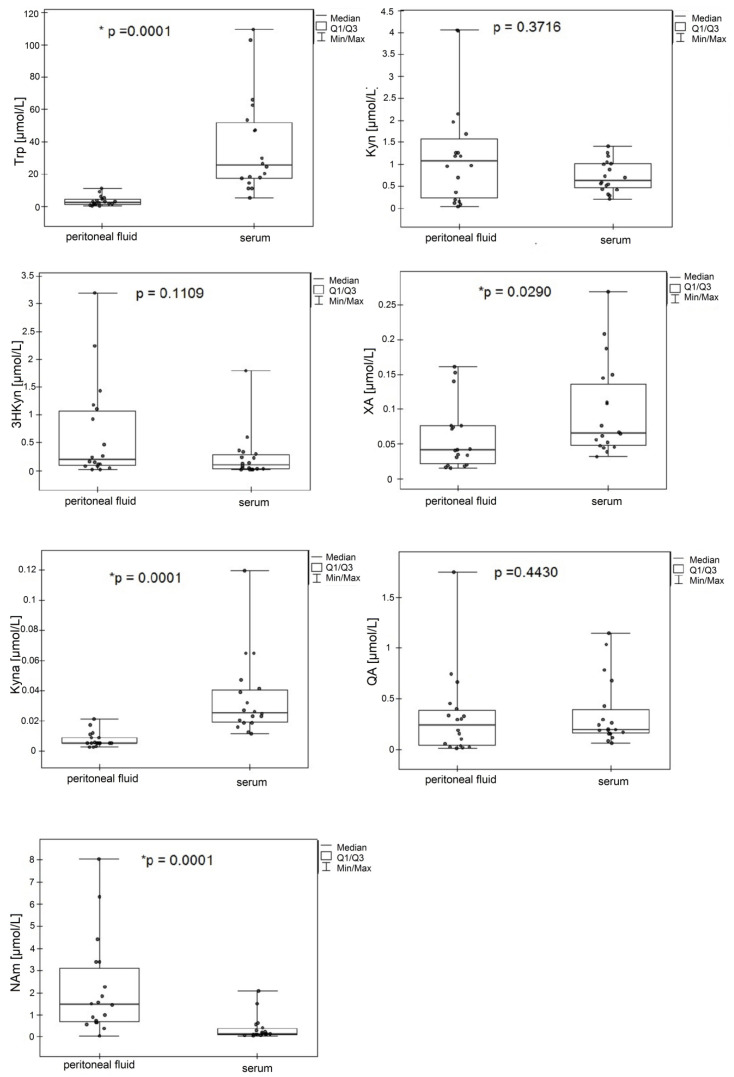
Comparison of concentrations of Trp, Kyn, 3HKyn, XA, Kyna, QA, and NAm determined in the serum and peritoneal fluid from GC patients (*n* = 18). * Statistically significant difference between concentration in the serum and peritoneal fluid.

The methods designed herein were applied for the determination of Trp, kynurenines, and NAm in the serum and peritoneal fluid from patients diagnosed with gastric cancer. The presented protocol allowed for simultaneous quantification of 8 and 10 different biomolecules in the serum and peritoneal fluid, respectively (Table 5). Since the serum was a very rich matrix, it was necessary to employ a protein precipitation step (using TCA) before chromatographic analysis. Unfortunately, the conditions were not compatible with the analysis of 3HAA and AA, unlike the peritoneal fluid samples, which were pretreated with acidified water and further purified by SPE. These two analytes were therefore determined only in the peritoneal fluid samples. The data were analyzed for all samples as the median values (Table 5). Differences between the concentrations of the selected analytes determined in the serum and peritoneal fluid are presented in Figure 4. As expected, we found a high amount of Trp in comparison with the KP metabolites in both matrices. Interestingly, the metabolites were found in the following abundance: Trp > Kyn > QA > Nam > 3HKyn > XA > Kyna in the serum or Trp > Nam > Kyn > 3HAA > QA > 3HKyn > XA > AA > Kyna in the peritoneal fluid. Kyn was the most prevalent metabolite, and Kyna was the least prevalent KP metabolite. There was also a different accumulation of Trp metabolites among the two analyzed matrices. While the Trp level was over ten times higher in the serum compared with the peritoneal fluid, the concentrations of Kyn, 3HKyn, and QA appeared to be higher in the peritoneal fluid. Nicotinamide (NAm), which is delivered with food and used up in the salvage pathway to form NAD+ (in a cascade of reactions involving QA as an intermediate), was found at over ten times a higher level in the peritoneal fluid than in the serum. Furthermore, higher concentrations of XA were reported in the serum than the peritoneal fluid. However, in this preliminary experiment, only for Trp, Kyna, XA and NAm were statistically significant differences between their concentrations in the serum and peritoneal fluid confirmed. All the tested peritoneal fluid samples contained 3HAA and AA at quantifiable nmol/L concentrations. The determined concentrations for Trp, Kyn, and Kyna in the serum were at similar levels compared to those reported by Choi et al. [24] for GC patients (3HKyn, QA, and NAm were not studied). This group also determined AA at an average concentration of 0.053 ± 0.049 µmol/mg/mL in the serum from GC patients.

## 3. Discussion

Indoleamine 2,3-dioxygenase (IDO) initializes tryptophan catabolism via the kynurenine pathway. IDO expression and Trp depletion are implicated in the molecular mechanisms of immune tolerance to foreign antigens in the tissue microenvironment [32]. Some KP metabolites exert immunological effects (e.g., suppression of T-cell proliferation in vitro, causing T-cell apoptosis and impairment of the natural killer (NK)-cell functions), causing dysregulation of the immune system and contributing to tumor-induced tolerance [32,33,34,35].

An increasing number of evidence suggests that IDO and metabolites of the KP pathway might contribute to GC progression or present novel therapeutic targets [17,18]. Kuligowski et al. identified Trp, Kyn, and phenylacetylglutamine as discriminant metabolites in GC [22]. Engin et al. found the correlation between a significantly higher serum Kyn-to-Trp ratio and increased neopterin levels in H. pylori seropositive GC patients [23]. Choi et al. evaluated Trp depletion via different metabolic routs in GC [24]. They determined the levels of Trp and its several metabolites (indole-3-lactic acid, AA, serotonin, nicotinic acid, Kyna, Kyn, and 3-indoxyl sulfate) in the serum and gastric juice of GC patients. The authors found activation of Trp metabolism via KP, showing that the Kyna level in serum and gastric juice might be considered a diagnostic indicator of GC.

Peritoneal dissemination is the most frequent metastasis site in GC [36,37,38,39] as it indicates disease progression. Since Trp metabolites are promising diagnostic targets in GC, we developed the UHPLC-ESI-MS/MS methods for Trp and kynurenines quantification in the serum and peritoneal fluid collected from GC patients. The challenge was the differential concentration of individual KP metabolites within one physiological matrix. Therefore, multi-target analysis required appropriate adjustment of the conditions for the sample preparation protocol, estimation of linear ranges, and proper instrument settings. Although there are LC-MS/MS methods available for determining the levels of Trp and its metabolites in serum [24,25,28,29,40], we presented here an alternative and validated protocol using one internal standard (3NT) and a simple sample preparation workflow. Furthermore, we described a protocol for quantification of Trp, NAm, and the biologically important KP metabolites (QA, 3HKyn, 3HAA, AA, Kyn, Kyna, and XA) in peritoneal fluid. This specimen was identified by us as an alternative material for Trp metabolite determination in GC patients. From an analytical standpoint, peritoneal fluid is a liquid containing sodium, potassium, chloride, phosphorus, calcium, bicarbonate, glucose, creatinine, uric acid, albumin, bilirubin, and other molecules [41]. According to some researchers, peritoneal fluid might be directly injected for LC-MS analysis [42]. Our samples were collected during surgical procedures (laparotomy and laparoscopy) and were yellowish to reddish in color, with fibers and other solids suggesting their complex composition. Our observations indicated that peritoneal fluid required pretreatment by SPE before LC-MS/MS to obtain reliable results and to avoid instrument contamination. While most studies focus on the metabolomics in blood, there is a lack of data available on the validated procedure for LC-MS/MS quantification of KP metabolites in the peritoneal fluid, especially in the context of gastric cancer. Pan et al. screened samples of peritoneal fluid from GC patients to identify differential metabolites, but no quantitative analysis of kynurenines has been performed [43].

In our approach, we also utilized an internal standard (3NT) at the beginning of the sample preparation step to allow correction for variations and errors throughout all procedure steps (e.g., volumetric variations and analyte loss during the procedure or matrix effect). Not endogenously present in the serum and peritoneal fluid was 3NT (Figure 3E,F), and it presented similar analytical behavior to the studied metabolites (Table 4). We previously tested the utility of 3NT as an internal standard for kynurenine quantification by LC-MS in post-culture media collected from human cancer cells, and the results were also satisfactory [30,44]. The linearity of the developed methods ranged from low amounts of nmol/L to tens of µmol/L. The estimated LOQs ranged from 2.45 to 23.00 nmol/L for the serum and 2.45 to 36.46 nmol/L for the peritoneal fluid (Table 2). Our protocol using TCA as a precipitation agent resulted in lower LOQs for Trp, Kyn, and Kyna in the serum, compared with the protocol developed by Choi et al. using ACN with formic acid [24], and for Trp, Kyn, Kyna, XA, QA, and 3HKyn, compared with the methodology designed in a 96-well plate format [28]. Galla et al. reported lower LOQs in serum for 3HKyn, XA, Kyna, and QA and higher LOQs for Trp and Kyn [29]. Lefèvre et al. reported lower LOQs for serum when working with standards prepared in a solvent and a Q-Exactive mass spectrometer, whereas our values were estimated in the presence of a sample matrix [40]. Regarding the LOQs for peritoneal fluid, we did not find other LC-MS/MS methods reported for simultaneous Trp, NAm, and kynurenine determination. Moreover, the proposed methodology allowed for the determination of NAm and several kynurenines in blood and peritoneal fluid, which were found to be within the µmol/L concentration levels (Figure 4).

Trp and kynurenine isotopes might share the same MRM transitions as the target analytes, interfering with their proper quantification [26,28]. The ^13^C-Trp isotope might share the transition selected for XA (206 > 160) in ESI+ mode. The ^13^C-Trp isotope (providing the [M + H]^+^ ion of theoretical *m/z* 206.1002) might be present at an abundance of 12.85% in relation to the ^12^C-Trp isotope. Thus, the monitored XA ion of *m/z* 206 (theoretical *m/z* 206.0448) might be enhanced by less than 13% by the presence of ^13^C-Trp isotopes in the sample. This interference might be important in the case of XA determination in biofluids (about 1000-fold and 100-fold Trp excess in relation to XA in the serum and peritoneal fluid, respectively; Table 5). However, with our chromatographic setup, these two molecules were well separated (RTs of 3.03 and 3.52 min for XA and Trp, respectively), and proper peak integration allowed for avoiding errors.

We applied the developed methods for NAm and KP metabolite monitoring in the serum and peritoneal fluid from GC patients (Table 5). This is the first demonstration of the major KP metabolites profile in a peritoneal fluid from cancer patients. It shows the utility of the developed methodology for the determination of Trp metabolites in the matrix, which is a major site of gastric cancer dissemination. Despite this being a preliminary study on samples from patients with different GC stages (based on pTNM), our results showed some important differences in the accumulation of Trp and its metabolites between the blood and peritoneal fluid, while statistically significant higher concentrations of Trp, Kyna, XA were found in serum, and NAm in the peritoneal fluid (Figure 4). The median concentrations of Kyn, 3HKyn, and QA were higher in the peritoneal fluid, but they were not statistically significant in this mixed patient population. We believe this is the first evidence of the possible importance of Trp metabolites locally in the cancer microenvironment that could be associated with the modulatory effect of these metabolites on an anti-cancer immune response. However, prediction of the relations of the distribution of Trp and its metabolites between the blood and peritoneal environments warrants extensive research. While this methodology was not suitable for the determination of 3HAA and AA in blood, we were able to quantify these metabolites in peritoneal fluid.

## 4. Materials and Methods

### 4.1. Reagents

Crystalline l-tryptophan (Trp, ≥98%), l-kynurenine (Kyn, ≥98%), 3-hydroxy-ᴅ,l-kynurenine (3HKyn), kynurenic acid (Kyna, ≥98%), nicotinamide (NAm, ≥99.5%), xanthurenic acid (XA, 96%), quinolinic acid (QA, 99%), 3-hydroxyanthranilic acid (3HAA), anthranilic acid (AA), 3-nitro-l-tyrosine (3NT), ammonium acetate (eluent additive for LC-MS), acetic acid (LC-MS grade), formic acid (LC-MS grade), trichloroacetic acid (TCA), activated charcoal, and bovine serum albumin (BSA) were purchased from Sigma-Aldrich (St. Louis, MO, USA). Methanol (MeOH, hypergrade), acetonitrile (ACN, hypergrade), hydrochloric acid (HCl), and ammonium hydroxide were acquired from Merck (Darmstadt, Germany). Stock solutions of Trp, Kyn, Kyna, XA, 3HAA, and AA were prepared by dissolving a reagent in dimethyl sulfoxide (DMSO, Merck, Darmstadt, Germany), 3HKyn in water acidified to pH 2.5 with HCl, NAm and QA in MeOH, and 3NT in 0.1% (*v*/*v*) formic acid in water. The stock solutions were stored at −20 °C and used for up to three freeze and thaw cycles. Working solutions at intermediate concentrations were prepared daily by dilution in methanol.

### 4.2. Instrumentation

Chromatographic measurements were carried out on a 1290 infinity ultra-high performance liquid chromatograph (UHPLC) consisting of a degasser, binary pump, autosampler and column thermostat (Agilent Technologies, Santa-Clara, CA, USA). The LC instrument set-up was coupled with an Agilent 6460 triple quadrupole mass spectrometer (QQQ) equipped with an electrospray ion source (Agilent Jet Stream). Data were acquired and analyzed with Agilent MassHunter Acquisition software v.B.08 and analyzed with Agilent MassHunter Quantitative Analysis software v.B.07. Chromatographic separation was achieved on the Agilent Technologies Zorbax Eclipse Plus C18 Rapid resolution HT column (2.1 × 100 mm, 1.8 µm) protected by a UHPLC Eclipse Plus C18 (2.1 mm, 1.8 µm) guard cartridge. Gradient elution was performed using a mobile phase consisting of 5 mmol/L aqueous ammonium acetate (solvent A) and MeOH (solvent B) with a column oven temperature at 40°C. The flow rate was set at 0.25 mL/min. For the serum samples, the elution gradient was programmed as follows: 0–5 min from 5% to 60% B; 5–6.5 min from 60% to 90% B; 6.5–7.5 min 90% B; 7.5–8.5 min from 90% to 5% B; and 8.5–12.0 min 5% B (post run: 2 min). For the peritoneal fluid samples, the elution gradient was programmed as follows: 0–5 min from 5% to 60% B; 5–6.5 min from 60% to 90% B; and 6.5–10 min 5% B (post run: 2 min). We used the following MS conditions: electrospray ionization in positive ion mode (ESI+); nebulizer gas: nebulizer 35 psi, gas temperature 300 °C, gas flow10 L/min, sheath gas temperature 300 °C, sheath gas flow10 L/min, and capillary voltage 4000 V. The mass spectrometer (MS) was operated in dynamic multiple reaction monitoring (DMRM) mode. An overview of the mass spectrometric conditions for each target compound is presented in Table 1.

The Isotope Distribution Calculator version 7.0.7024.0 and Agilent MassHunter Workstation Data Analysis Core software were used for estimation of the abundances of different Trp and XA isotopes. For calculations, the +1 charge of the molecule, which corresponded to [M + H]^+^, was selected.

For reagent and sample preparation, an XP6 microbalance (Mettler Toledo, Switzerland), 5415R and 5804 centrifuges (Eppendorf, Germany), a SevenMulti^™^ dual meter pH/conductivity completed with InLab^®^ Expert Pro (Mettler Toledo, Switzerland), a Stuart SSL4 see-saw rocker, and anEZ-2 Elite Personal Evaporator (Genevac Ltd., Ipswich, UK) were used. Ultrapure water was generated with a Milli-Q water purification system (Merck Millipore, Darmstadt, Germany). Solid-phase extraction (SPE) was carried out using the Strata-X columns (Polymeric Reversed Phase, surface modified styrene divinylebenzene; particle size: 33 µm, 30 mg/1 mL; pore size: 85 Å; pore volume: 1.2 mL/g; surface area: 800 m^2^/g) with columns supplied by Phenomenex (Aschaffenburg, Germany) and a Gilson GX-271 ASPEC (Middleton, WI, USA) automatic extraction system operated by Gilson Ethernet Utility 1.8.6.1 and Trilution LH 2.0 software. SPE cartridges SOLA HRP and SOLA SCX (10 mg/1 mL) from Thermo Fisher Scientific (Waltham, MA, USA) were also tested.

### 4.3. Patient Characteristics

Our study included 18 patients admitted to the Department of Surgical Oncology at the Medical University of Lublin, Poland with advanced gastric adenocarcinoma (pT2-4, pN0-3, pM0-1) diagnosed based on abdominal and pelvic computed tomography (CT) diagnostic imaging and cytology performed on the peritoneal fluid according to the routine diagnostic procedure. There were 10 males (age 41–80 years) and 8 females (age 45−81 years). Peritoneal fluid samples were collected during laparoscopy or surgical procedure. Serum was obtained from whole blood samples by centrifugation at 6000× *g* for 15 min at room temperature and separation of a cellular clot. All samples were stored frozen at −80 °C until analysis. This study was approved by the Bioethical Committee of the Medical University of Lublin (ethic code: KE–0254/182/2018 from 28.06.2018).

### 4.4. Preparation of Serum Samples

Prior to analysis, the serum samples were left to thaw at room temperature. Then, 95 µL of serum was transferred into a conical tube, mixed with 5 µL of the internal standard (44.2 µmol/L 3NT) and vortexed thoroughly. Then, 50 µL of 15% (*w*/*v*) TCA solution was added for protein precipitation. After vortexing, the samples were centrifuged (14,000× *g*, 15 min, 4 °C). The collected supernatants were recentrifuged, and the clear aliquot was transferred into the chromatographic insert vial and immediately analyzed in triplicate by the UHPLC-ESI-MS/MS method (the injection volume was 10 µL). For Trp determination, 47.5 µL of serum was fortified with 2.5 µL of the internal standard (4.42 mmol/L 3NT), vortexed well, mixed with 25 µL of 15% (*w*/*v*) of the TCA solution, and vortexed once again. After centrifugation (14,000× *g*, 15 min, 4 °C), the collected supernatants were recentrifuged. A clear supernatant was diluted 100times with a 5-mmol/L aqueous solution of ammonium acetate and analyzed in triplicate by the UHPLC-ESI-MS/MS method.

### 4.5. Preparation of Peritoneal Fluid

A sample of the peritoneal fluid (395 µL) was mixed with 5 µL of the internal standard solution (4.42 mmol/L 3NT) and 500 µL of cold 1% (*v*/*v*) acetic acid in water. After vortexing, the samples were centrifuged (14,000× *g*, 15 min, 4 °C), and the collected supernatant was further purified by SPE. The SPE cartridges were conditioned and equilibrated with 1 mL of MeOH and 1 mL of water, respectively. Then, 500 µL of the sample was loaded onto the cartridge, which was further washed with 1 mL of water. Elution was carried out with 1 mL of the mixture of MeOH/ACN/3 mol/L acetic acid (40:40:20, *v*/*v*/*v*). The eluate was transferred into a glass vial and dried using an evaporator. The residue was redissolved in 100 µL of 5 mmol/L ammonium acetate under sonication (5 min). When the sample contained some debris, it was centrifuged (14,000× *g*, 15 min, 4 °C), and the clear supernatant was transferred into a glass chromatographic insert vial and immediately analyzed in triplicate by the UHPLC-ESI-MS/MS method. The injection volume was 5 µL. The samples were concentrated or diluted if needed to fit the linear range of the calibration curves.

### 4.6. Preparation of the Charcoal-Pretreated Serum and Peritoneal Fluid

The serum and peritoneal fluid were pretreated with activated charcoal to remove Trp and its metabolites from the biological matrix, which were to be used for matrix-matched calibration. Based on the previously described protocols [30,44,45], the activated charcoal (280 mg) was mixed with 5 mL of the serum or peritoneal fluid and shaken (50 osc./min) for 2 h at room temperature. After centrifugation (15 min, 6000× *g*), the supernatant was mixed with a fresh portion of charcoal (280 mg), shaken (50 osc./min, 2 h, room temperature), and centrifuged (15 min, 6000× *g*). The final supernatant was filtered on a syringe filter (regenerated cellulose, 0.45 µm) and stored at 4 °C until analysis.

### 4.7. Preparation of the Matrix-Matched Calibration Standards

In the case of the serum, for the method’s calibration and validation, a BSA solution in ultrapure water (40 g/L) was used as a surrogate matrix. The reference sample of the peritoneal fluid was obtained by mixing samples delivered from at least from five gastric cancer patients. Matrix-matched calibration solutions, quality control (QC) samples were prepared using charcoal-purified serum or peritoneal fluid (protocol provided above). The quality control (QC) standards consisted of a mixture prepared from the working solutions, containing target analytes at four different concentration levels (LOQ, low, medium, and high) and a constant amount of 3NT. The concentrations of the QC samples are summarized in Table 1.

### 4.8. Method Validation

#### 4.8.1. Limit of Detection, Limit of Quantification, and Linearity

Matrix-matched curves were built using charcoal-pretreated matrices. The calibration solutions contained fixed amounts of the internal standard (3NT) and at least six different concentrations of analytes. Each sample was run in triplicate. The blank samples (spiked with 3NT) were also analyzed (if needed, the obtained values were subtracted from each calibration point). The calibration solutions were processed as described in the section on sample preparation. The regression plots were obtained using the ratio of the peak area for the analyte of interest over the 3NT peak area versus the analyte concentration. The calibration curves were built in at least three different days. The limit of detection (LOD) and quantification (LOQ) were determined from the matrix-matched calibration curves as a standard deviation of the intercepts (*n* = 3) divided by the slope of the calibration function and multiplied by 3.3 or 10, respectively. The calibration curves’ linearities were established by the coefficient of determination (R^2^ of 0.99 or greater was required).

#### 4.8.2. Precision and Accuracy

The precision and accuracy were determined by analyzing the QC samples at the LOQ, low (LOQ × 3), medium (corresponding to the medium concentration from the calibration plot), and high (the highest point of the calibration curve) concentration levels in both matrices independently. The QC concentrations are detailed in Table 3. The precision was expressed by the coefficient of variation (CV). The accuracy was obtained by dividing the obtained mean concentration by a nominal value and expressing it as a percentage. The interday precision and accuracy were determined on the same day by six repeated measurements. The measurements for the intraday precision and accuracy were performed on three different days. The acceptable limits of the precision and accuracy were ±20% at LOQ and ±15% at other concentration levels.

#### 4.8.3. Recovery, Matrix Effects, and Stability

The recoveries, matrix effects, and stability for both matrices were evaluated at four different levels of analytes from the calibration range (LOQ, low, medium, and high). The corresponding concentrations of the QC standards are summarized in Table 4. The concentration of the internal standard (3NT) was constant in all the QC samples. The analysis was performed on the following samples: (1) standards prepared in 5 mmol/L ammonium acetate (sample A), (2) charcoal-pretreated serum or peritoneal fluid spiked before being subjected to the sample preparation protocol (sample B), and (3) charcoal-pretreated serum or peritoneal fluid spiked at the end of the sample preparation step (sample C). The recoveries and matrix effects were calculated using Equations (1) and (2), respectively. The stability of the analytes in the QC standards was tested at room temperature in the autosampler tray after 24 h of storage and calculated with Equation (3). The freeze–thaw stability of the QC standards was evaluated after three cycles of freezing (at −20 °C) and thawing (at room temperature) and estimated according to Equation (4). Each sample was analyzed six times:

(1) $$\mathrm{Recovery}\;\left[\%\right]=\frac{\mathrm{Analyte}\;\mathrm{peak}\;\mathrm{area}\;\mathrm{in}\;\mathrm{sample}\;B}{\mathrm{Analyte}\;\mathrm{peak}\;\mathrm{area}\;\mathrm{in}\;\mathrm{sample}\;C}\times 100\%$$

(2) $$\mathrm{Matrix}\;\mathrm{effect}\;\left[\%\right]=\frac{\mathrm{Analyte}\;\mathrm{peak}\;\mathrm{are}\;\mathrm{in}\;\mathrm{sample}\;C}{\mathrm{Analyte}\;\mathrm{peak}\;\mathrm{area}\;\mathrm{in}\;\mathrm{sample}\;A}\times 100\%$$

(3) $$\mathrm{Stability}\;\left[\%\right]=\frac{\mathrm{Analyte}\;\mathrm{peak}\;\mathrm{area}\;\mathrm{in}\;\mathrm{freshly}\;\mathrm{prepared}\;\mathrm{sample}\;B\;}{\mathrm{Analyte}\;\mathrm{peak}\;\mathrm{area}\;\mathrm{after}\;24\;h}\times 100\%$$

(4) $$\mathrm{Freeze}-\mathrm{thaw}\;\mathrm{stability}\;\left[\%\right]=\frac{\mathrm{Analyte}\;\mathrm{peak}\;\mathrm{area}\;\mathrm{in}\;\mathrm{freshly}\;\mathrm{prepared}\;\mathrm{sample}\;B\;}{\mathrm{Analyte}\;\mathrm{peak}\;\mathrm{area}\;\mathrm{after}\;\mathrm{freezing}\;\mathrm{and}\;\mathrm{thawing}}\times 100\%$$

### 4.9. Statistical Analysis

PQStat software v.1.8.2.160 was used for statistical analysis. Data normality was evaluated by the Shapiro–Wilk test. Thus, a normal distribution was not confirmed, and comparison of the analyte concentrations determined in the samples of serum and peritoneal fluid were performed using the Mann–Whitney U Test. Statistical significance was accepted at *p* < 0.05.

## 5. Conclusions

Early diagnosis of GC significantly extends patients’ survival. Identification of the markers for GC progression will allow for developing a screening test to identify high-risk patients and reduce their mortality rate. Therefore, the development of accurate analytical methods for biomarker detection and quantification has become an active field of research. Kynurenine pathway metabolites are suggested to be important prognostic factors of gastric cancer. Herein, the described UHPLC-ESI-MS/MS approach for quantification of Trp, NAm, and KP metabolites has been developed for application in serum and peritoneal fluid analysis. Using this method in further studies on the correlation between NAm and kynurenine accumulation in different body fluids in GC patients might bring about novel biomarkers for the prediction of disease progression.

## Figures and Tables

**Table 1:** Selected DMRM transitions and instrument settings.

Analyte	Precursor ion (*m*/*z*)	Product ion (*m*/*z*)	FV (V)	CE (eV)	RT (min)	ΔRT	Polarity
QA	168.0	150.0	60	8	1.10	1.50	ESI+
		124.0	60	8			
3HKyn	225.0	208.0	80	8	1.72	1.50	ESI+
		162.0	80	10			
3HAA *	154.0	136.0	100	8	1.20	1.50	ESI+
		103.0	100	20			
AA *	138.0	120.0	100	4	2.32	1.50	ESI+
		91.5	100	16			
NAm	123.0	96.0	110	20	2.58	2.00	ESI+
		106.0	110	20			
Kyn	209.0	192.0	100	8	2.64	2.00	ESI+
		174.0	100	10			
3NT **	227.0	181.0	80	8	2.80	2.00	ESI+
		117.0	80	20			
XA	206.0	160.0	110	20	3.03	2.00	ESI+
		188.0	110	8			
Kyna	190.0	144.0	100	20	3.32	2.00	ESI+
		172.0	100	10			
Trp	205.0	188.0	110	8	3.52	2.00	ESI+
		159.0	110	10			

* Determined only in samples of peritoneal fluid. ** Internal standard. QA: quinolinic acid; 3HAA: 3-hydroxyanthranilic acid; AA: anthranilic acid; 3HKyn: 3-hydroxykynurenine; NAm: nicotinamide; Kyn: kynurenine; Kyna: kynurenic acid; Trp: tryptophan; XA: xanthurenic acid; 3NT: 3-nitrotyrosine; FV: fragmentor voltage; CV: collision energy; RT: retention time.

**Table 2:** Linearity and parameters of the calibration curves.

	Serum						Peritoneal Fluid					
Analyte	Calibration Range (µmol/L)	LOD (nmol/L)	LOQ (nmol/L)	Regression Parameters			Calibration Range (µmol/L)	LOD (nmol/L)	LOQ (nmol/L)	Regression Parameters		
				Slope	Intercept	R^2^				Slope	Intercept	R^2^
QA	0.01–2.99	3.95	11.96	0.117	−0.004	0.998	0.02–11.96	5.92	17.94	0.017	0.001	0.993
3HKyn	0.02–2.23	7.59	23.00	0.045	−0.001	0.999	0.02–9.20	7.59	23.00	0.004	0.001	0.999
3HAA	n.s.	n.s.	n.s.	n.s.	n.s.	n.s.	0.03–13.06	10.77	32.65	0.013	0.001	0.999
AA	n.s.	n.s.	n.s.	n.s.	n.s.	n.s.	0.0–14.58	12.03	36.46	0.067	−0.018	0.994
NAm	8.19 *–4.10	2.70	8.19	0.118	−0.001	0.998	8.19 *–16.38	2.70	8.19	0.013	0.008	0.996
Kyn	4.80 *–2.40	1.58	4.80	1.225	0.013	0.999	4.80 *–9.60	1.58	4.80	0.096	0.030	0.997
XA	0.01–1.47	3.23	9.80	3.599	0.029	0.995	0.01–9.50	4.70	14.25	0.704	0.001	0.999
Kyna	5.30 *–2.65	1.75	5.30	10.732	0.012	0.999	5.32 *–10.64	1.76	5.32	1.572	0.230	0.998
Trp	2.45 *–2.45	0.81	2.45	3.257	0.002	0.998	2.45 *–9.80	0.81	2.45	0.461	0.353	0.998

n.s.: not studied; LOD: limit of detection; LOQ: limit of quantification; R^2^: coefficient of determination. * values in nmol/L.

**Table 3:** Precision and accuracy of the methods.

	Serum							Peritoneal Fluid						
Analyte	Spiked (µmol/L)	Intraday (*n* = 6)			Interday (*n* = 18)			Spiked (µmol/L)	Intraday (*n* = 6)			Interday (*n* = 18)		
		Precision		Accuracy (%)	Precision		Accuracy (%)		Precision		Accuracy (%)	Precision		Accuracy (%)
		Found ± SD (µmol/L)	CV (%)		Found ± SD (µmol/L)	CV (%)			Found ± SD (µmol/L)	CV (%)		Found ± SD (µmol/L)	CV (%)	
QA	11.96 *	12.96 ± 0.36 *	2.90	108.35	11.99 ± 1.57 *	13.11	100.25	17.94 *	21.10 ± 2.79 *	13.19	117.95	20.51 ± 3.20 *	15.60	114.33
	35.88 *	37.71 ± 2.15 *	5.71	105.11	37.4 ± 2.11 *	5.92	104.39	53.82 *	58.4 ± 7.66 *	13.10	108.68	57.93 ± 7.85 *	13.56	107.64
	1.50	1.56 ± 0.08	4.98	104.23	1.45 ± 0.18	12.10	96.69	5.98	6.42 ± 0.20	3.12	107.37	6.21 ± 0.83	13.33	103.84
	2.99	2.74 ± 0.06	2.05	91.71	2.69 ± 0.37	13.62	89.84	11.96	12.2 ± 0.34	2.76	102.11	11.60 ± 0.70	6.05	96.97
3HKyn	23.00 *	20.00 ± 2.36 *	11.78	86.95	19.8 ± 3.12 *	15.72	86.24	23.00 *	20.8 ± 3.41 *	16.37	90.47	20.59 ± 2.95 *	14.31	89.51
	111.50 *	98.02 ± 6.24 *	6.36	87.91	97.5 ± 9.84 *	10.08	87.52	69.00 *	61.8 ± 5.23 *	8.46	89.58	62.71 ± 6.67 *	10.64	90.89
	1.12	1.06 ± 0.06	5.28	94.72	1.04 ± 0.13	12.58	92.83	2.30	2.20 ± 0.27	12.37	95.67	2.15 ± 0.24	11.36	93.57
	2.23	2.31 ± 0.16	7.07	103.78	2.25 ± 0.26	11.33	100.96	9.20	8.07 ± 0.26	3.19	87.55	7.95 ± 0.48	6.08	86.37
3HAA		n.s.	n.s.	n.s.	n.s.	n.s.	n.s.	32.65 *	36.80 ± 4.01 *	10.88	112.94	34.77 ± 4.41 *	12.64	106.49
		n.s.	n.s.	n.s.	n.s.	n.s.	n.s.	97.95 *	97.60 ± 7.57 *	7.76	99.64	98.11 ± 8.46 *	8.62	100.16
		n.s.	n.s.	n.s.	n.s.	n.s.	n.s.	3.27	3.41 ± 0.37	10.98	104.40	3.27 ± 0.47	14.39	99.23
		n.s.	n.s.	n.s.	n.s.	n.s.	n.s.	13.06	13.90 ± 0.73	5.26	106.54	14.36 ± 0.89	6.20	109.25
AA		n.s.	n.s.	n.s.	n.s.	n.s.	n.s.	36.46 *	30.60 ± 1.60 *	5.21	83.90	30.81 ± 2.62 *	8.50	84.41
		n.s.	n.s.	n.s.	n.s.	n.s.	n.s.	109.40 *	104.78 ± 4.62 *	4.41	95.77	109.40 ± 10.25 *	9.37	100.03
		n.s.	n.s.	n.s.	n.s.	n.s.	n.s.	3.65	3.66 ± 0.27	7.24	100.31	3.77 ± 0.24	6.27	103.30
		n.s.	n.s.	n.s.	n.s.	n.s.	n.s.	14.58	15.03 ± 0.47	3.09	103.06	4.45 ± 1.31	9.06	99.06
NAm	8.19 *	7.13 ± 1.04 *	14.58	87.11	7.61 ± 1.36 *	17.81	92.96	8.19 *	8.13 ± 1.12 *	13.73	99.22	8.62 ± 1.26 *	14.57	105.29
	24.60 *	23.86 ± 1.29 *	5.41	96.98	24.5 ± 1.47 *	5.98	99.97	24.60 *	26.75 ± 2.61 *	9.76	108.88	25.64 ± 2.96 *	11.54	104.35
	2.05	2.27 ± 0.05	2.19	110.77	2.35 ± 0.12	5.25	114.44	4.10	4.02 ± 0.60	14.95	98.26	3.89 ± 0.37	9.57	94.99
	4.10	4.65 ± 0.25	5.31	113.34	4.67 ± 0.21	4.45	113.80	16.38	16.16 ± 0.68	4.21	98.66	16.35 ± 1.46	8.92	99.84
Kyn	4.80 *	4.60 ± 0.38 *	8.28	95.86	4.57 ± 0.77 *	16.92	95.30	4.80 *	4.48 ± 0.64 *	14.34	93.36	4.66 ± 0.50 *	10.66	97.11
	14.40 *	15.14 ± 0.79 *	5.21	105.13	14.5 ± 1.30 *	8.89	101.23	14.40 *	14.28 ± 1.24 *	8.66	99.18	14.11 ± 1.37 *	9.69	97.96
	1.20	1.25 ± 0.03	2.60	103.96	1.34 ± 0.20	14.86	111.81	2.40	2.35 ± 0.08	3.38	97.80	2.29 ± 0.14	6.19	95.58
	2.40	2.50 ± 0.09	3.78	104.26	2.74 ± 0.22	8.19	114.11	9.60	8.84 ± 0.36	4.07	92.03	9.22 ± 0.59	6.42	96.37
XA	9.80 *	10.62 ± 0.58 *	5.48	108.32	10.4 ± 0.73 *	6.98	106.39	14.25 *	15.36 ± 1.11 *	7.25	107.79	15.13 ± 1.33 *	8.77	106.16
	29.40 *	33.22 ± 1.49 *	4.49	112.98	33.7 ± 2.47 *	7.32	114.68	42.75 *	44.52 ± 5.27 *	11.84	104.14	42.33 ± 5.58 *	13.18	99.03
	0.74	0.83 ± 0.03	3.21	112.57	0.77 ± 0.10	13.03	104.53	2.38	2.22 ± 0.12	5.64	93.17	2.25 ± 0.22	9.97	94.57
	1.47	1.63 ± 0.04	2.28	110.67	1.58 ± 0.18	11.31	107.47	9.50	10.51 ± 0.45	4.24	110.67	9.54 ± 1.25	13.11	100.46
Kyna	5.30 *	6.03 ± 0.19 *	3.08	113.76	6.09 ± 0.18 *	3.04	114.84	5.32 *	4.95 ± 0.54 *	10.98	93.03	5.31 ± 0.76 *	14.24	99.75
	31.80 *	34.79 ± 2.05 *	5.90	109.40	34.6 ± 2.35 *	6.77	109.03	15.96 *	16.93 ± 1.49 *	8.79	106.06	16.69 ± 1.98 *	11.88	104.60
	1.33	1.37 ± 0.03	2.11	103.05	1.43 ± 0.08	5.87	107.72	2.66	2.44 ± 0.15	6.24	91.63	2.65 ± 0.19	7.14	99.68
	2.65	3.01 ± 0.11	3.75	113.72	3.04 ± 0.09	2.97	114.76	10.64	11.50 ± 0.52	4.54	108.05	10.49 ± 0.88	8.38	98.61
Trp	2.45 *	2.30 ± 0.23 *	10.09	94.04	2.34 ± 0.25 *	10.82	95.57	2.45 *	2.45 ± 0.25 *	9.88	101.03	2.45 ± 0.38 *	15.53	100.15
	7.35 *	8.29 ± 0.54 *	6.53	112.84	8.19 ± 0.99 *	12.05	111.40	7.35 *	7.19 ± 0.56 *	7.81	97.88	7.93 ± 0.80 *	10.05	107.84
	1.23	1.38 ± 0.06	4.55	113.72	1.40 ± 0.05	3.47	113.73	2.45	2.28 ± 0.22	9.83	93.23	2.42 ± 0.27	11.08	98.68
	2.45	2.61 ± 0.08	3.02	106.41	2.74 ± 0.11	4.09	111.94	9.80	8.92 ± 0.34	3.77	91.06	8.94 ± 0.42	4.68	91.21

* Values in nmol/L; n.s.: not studied.

**Table 4:** Recovery, matrix effects, and stability.

	Serum					Peritoneal Fluid				
Analyte	Spiked (µmol/L)	RE ± SD (%)	ME ± SD (%)	Stability ** ± SD (%)	FT Stability ± SD (%)	Spiked (µmol/L)	RE± SD (%)	ME ± SD (%)	Stability ** ± SD (%)	FT Stability ± SD (%)
QA	11.96 *	83.75 ± 2.44	129.56 ± 5.64	95.45 ± 9.78	84.16 ± 3.68	17.94 *	82.97 ± 3.76	73.90 ± 3.96	96.02 ± 12.45	97.99 ± 9.91
	35.88 *	95.16 ± 3.14	104.40 ± 6.46	92.75 ± 12.26	93.00 ± 7.13	53.82 *	87.94 ± 1.66	86.12 ± 4.23	92.40 ± 8.55	101.09 ± 9.15
	1.50	86.50 ± 3.36	97.39 ± 6.94	98.41 ± 1.03	121.28 ± 1.27	5.98	89.58 ± 2.52	84.23 ± 7.67	96.14 ± 7.86	99.01 ± 10.52
	2.99	94.86 ± 1.40	109.93 ± 1.36	102.33 ± 1.12	105.38 ± 1.50	11.96	90.06 ± 4.02	83.74 ± 8.22	98.71 ± 8.39	100.16 ± 9.37
3HKyn	23.00 *	92.90 ± 7.35	80.92 ± 10.84	76.28 ± 12.62	79.62 ± 5.28	23.00 *	49.39 ± 5.29	89.90 ± 2.86	87.87 ± 6.64	70.60 ± 9.90
	111.50 *	88.25 ± 8.00	109.19 ± 11.57	53.74 ± 10.14	73.73 ± 5.16	0.07	50.40 ± 2.76	81.81 ± 2.28	87.42 ± 6.69	69.10 ± 8.74
	1.12	87.95 ± 7.31	87.15 ± 7.04	65.51 ± 7.14	70.90 ± 12.56	2.30	50.99 ± 2.06	76.59 ± 3.59	83.35 ± 4.25	64.28 ± 4.95
	2.23	100.35 ± 6.45	94.17 ± 8.72	64.22 ± 6.92	76.46 ± 13.13	9.20	57.89 ± 4.41	77.43 ± 8.77	83.70 ± 8.71	64.87 ± 6.41
3HAA		n.s.	n.s.	n.s.	n.s.	32.65 *	91.84 ± 2.74	80.40 ± 8.95	101.12 ± 13.82	94.02 ± 6.12
		n.s.	n.s.	n.s.	n.s.	97.95 *	97.18 ± 9.22	85.88 ± 7.10	101.15 ± 7.27	95.61 ± 3.21
		n.s.	n.s.	n.s.	n.s.	3.27	108.89 ± 4.89	85.10 ± 3.54	67.71 ± 7.28	90.39 ± 8.15
		n.s.	n.s.	n.s.	n.s.	13.06	112.70 ± 3.63	88.10 ± 2.78	66.82 ± 5.35	93.15 ± 4.99
AA		n.s.	n.s.	n.s.	n.s.	36.46 *	95.66 ± 9.13	77.37 ± 6.44	97.89 ± 9.93	94.42 ± 3.99
		n.s.	n.s.	n.s.	n.s.	109.40 *	96.89 ± 5.37	80.58 ± 2.77	104.84 ± 7.46	105.06 ± 5.63
		n.s.	n.s.	n.s.	n.s.	3.65	89.98 ± 3.40	80.80 ± 9.16	92.88 ± 2.18	100.43 ± 12.91
		n.s.	n.s.	n.s.	n.s.	14.58	90.42 ± 2.02	87.32 ± 4.10	88.95 ± 2.76	97.18 ± 4.49
NAm	8.19 *	91.92 ± 8.56	113.53 ± 7.41	105.99 ± 9.19	76.83 ± 12.19	8.19 *	83.64 ± 7.05	126.55 ± 6.53	140.51 ± 15.84	100.07 ± 7.40
	24.60 *	93.73 ± 5.63	104.40 ± 6.46	107.76 ± 9.21	108.47 ± 7.48	24.60 *	85.47 ± 6.10	114.41 ± 9.97	128.61 ± 16.89	97.60 ± 6.57
	2.05	97.61 ± 3.52	121.20 ± 3.49	102.18 ± 1.58	98.89 ± 2.51	4.10	85.89 ± 3.46	128.01 ± 1.75	104.55 ± 6.60	103.33 ± 5.93
	4.10	96.98 ± 3.89	154.05 ± 10.24	102.56 ± 1.90	99.62 ± 0.86	16.38	85.45 ± 2.08	107.11 ± 6.94	107.93 ± 4.79	104.57 ± 9.49
Kyn	4.80 *	93.61 ± 5.75	100.75 ± 3.88	105.73 ± 1.85	90.93 ± 3.35	4.80 *	85.53 ± 3.33	123.75 ± 8.17	112.48 ± 10.58	98.15 ± 9.62
	14.40 *	103.62 ± 7.72	109.87 ± 6.52	108.02 ± 11.62	107.62 ± 2.91	14.40 *	86.33 ± 6.10	122.74 ± 9.64	109.01 ± 12.78	98.01 ± 3.71
	1.20	91.11 ± 3.65	125.61 ± 4.40	110.11 ± 3.18	108.58 ± 0.52	2.40	88.78 ± 1.27	121.38 ± 11.30	91.92 ± 4.47	105.19 ± 6.35
	2.40	96.40 ± 1.75	133.20 ± 9.63	102.17 ± 0.79	109.16 ± 0.84	9.60	94.43 ± 3.38	122.84 ± 10.86	97.62 ± 6.84	101.01 ± 5.10
XA	9.80 *	81.44 ± 3.58	84.55 ± 7.12	124.49 ± 7.22	126.20 ± 7.64	14.25 *	103.43 ± 8.60	113.55 ± 5.38	92.28 ± 6.76	83.76 ± 6.29
	29.40 *	87.09 ± 7.31	72.26 ± 2.98	90.99 ± 6.38	93.19 ± 5.58	42.75 *	109.06 ± 8.95	115.42 ± 1.22	103.87 ± 4.20	90.31 ± 8.12
	0.74	85.17 ± 4.16	78.61 ± 8.25	103.26 ± 1.06	99.01 ± 5.82	2.38	110.33 ± 8.66	97.24 ± 4.25	82.58 ± 5.39	87.07 ± 7.58
	1.47	86.13 ± 3.62	79.80 ± 4.61	99.10 ± 5.12	99.63 ± 1.71	9.50	111.00 ± 4.33	100.46 ± 1.84	84.56 ± 5.26	84.43 ± 8.02
Kyna	5.30 *	91.82 ± 2.73	83.76 ± 1.44	98.64 ± 2.46	111.62 ± 4.28	5.32 *	87.88 ± 5.18	119.91 ± 10.49	140.90 ± 14.55	92.32 ± 6.14
	31.80 *	98.41 ± 6.58	95.35 ± 2.27	107.32 ± 6.25	109.01 ± 7.80	15.96 *	91.94 ± 7.02	113.44 ± 11.32	131.72 ± 14.84	97.08 ± 3.90
	1.33	99.59 ± 2.79	91.74 ± 7.54	103.74 ± 1.88	107.67 ± 0.79	2.66	108.88 ± 4.60	105.65 ± 0.98	98.89 ± 3.61	103.82 ± 4.67
	2.65	101.85 ± 1.32	90.71 ± 0.34	102.10 ± 1.12	106.99 ± 0.30	10.64	113.62 ± 3.33	103.96 ± 1.97	92.33 ± 3.58	96.53 ± 1.58
Trp	2.45 *	88.27 ± 2.92	86.76 ± 2.61	92.76 ± 4.05	97.55 ± 3.02	2.45 *	95.29 ± 6.37	114.70 ± 11.78	123.18 ± 6.03	98.17 ± 3.72
	7.35 *	88.10 ± 7.12	66.07 ± 6.20	88.21 ± 10.57	89.20 ± 3.45	7.35 *	94.51 ± 2.85	97.32 ± 11.01	118.11 ± 5.61	98.86 ± 6.53
	1.23	88.96 ± 2.61	71.47 ± 4.16	87.70 ± 3.24	99.79 ± 2.31	2.45	99.12 ± 1.46	93.09 ± 3.63	96.63 ± 8.19	104.80 ± 6.49
	2.45	87.51 ± 2.48	74.50 ± 1.15	89.94 ± 3.18	99.44 ± 0.15	9.80	115.35 ± 3.83	101.14 ± 3.95	104.82 ± 4.51	109.17 ± 9.42
3NT	2.21	98.23 ± 2.55	101.82 ± 2.27	101.15 ± 5.33	98.20 ± 1.70	55.25	104.97 ± 2.51	97.19 ± 4.85	81.14 ± 4.75	89.54 ± 4.01

* Values in nmol/L. ** Stability after keeping samples in an autosampler for 24 h. SD: standards deviation (*n* = 6); n.s.: not studied; RE: recovery; ME: matrix effect; FT Stability: freeze–thaw stability.

**Table 5:** Concentration ranges of Trp, NAm, and KP metabolites in the serum and peritoneal fluid from gastric cancer patients (*n* = 18).

Analyte	Serum (µmol/L)			Peritoneal Fluid (µmol/L)		
	Min	Median	Max	Min	Median	Max
Trp *	5.15	25.64	109.56	0.34	2.35	11.22
Kyn	0.20	0.64	1.41	0.04	1.07	4.06
3HKyn	0.02	0.11	1.80	0.02	0.20	3.20
XA *	0.03	0.07	0.27	0.02	0.04	0.16
Kyna *	0.01	0.03	0.12	2.52 **	5.23 **	21.04 **
AA	n.s.	n.s.	n.s.	0.02	0.04	0.43
3HAA	n.s.	n.s.	n.s.	0.16	0.43	2.03
QA	0.07	0.20	0.15	0.01	0.24	1.75
NAm *	0.04	0.13	2.09	0.04	1.47	8.04

n.s.: not studied. * Statistically significant difference between concentration in the serum and peritoneal fluid. ** Values in nmol/L.
